# microRNA-100 Targets SMRT/NCOR2, Reduces Proliferation, and Improves Survival in Glioblastoma Animal Models 

**DOI:** 10.1371/journal.pone.0080865

**Published:** 2013-11-14

**Authors:** Bahauddeen M. Alrfaei, Raghu Vemuganti, John S. Kuo

**Affiliations:** 1 Department of Neurological Surgery and Cellular and Molecular Pathology Training Program, University of Wisconsin School of Medicine and Public Health, Madison, Wisconsin, United States of America; 2 Departments of Neurological Surgery and Human Oncology, Cellular and Molecular Pathology Training Program, and Carbone Cancer Center, University of Wisconsin School of Medicine and Public Health, Madison, Wisconsin, United States of America; Schulze Center for Novel Therapeutics, Mayo Clinic, United States of America

## Abstract

Glioblastoma (GBM) is the most frequently diagnosed malignant human glioma, and current median patient survival is less than two years despite maximal surgery followed by temozolomide chemoradiation therapies. Novel microRNA-related therapies are now being developed for cancers such as GBM. Differential microRNA expression profiling revealed that miR-100 expression is down-regulated in GBM compared to normal controls. We report that miR-100 expression reduces GBM tumorigenicity. In vitro, four GBM lines (U87, U251, 22T, and 33T) demonstrated reduced proliferation 24 hours after transient miR100 overexpression via transfection. miR-100 triggered cell death an average 70% more than scrambled miR controls 24 hours after transient transfection (p < 0.01). miR-100 targeted inhibition of the “silencing mediator of retinoid or thyroid hormone receptor-2” (SMRT/NCOR2) gene was confirmed via reporter assays. Ki67 proliferation index was decreased 40% in tumor xenografts generated from stable miR-100 transfected GBM lines versus controls (p < 0.01). Furthermore, treatment of tumor xenografts with a single pre-mir-100 injection (60 pmol) significantly extended survival of mice bearing intracranial GBM xenografts 25% more than scrambled controls (p < 0.01; n=8). These studies establish miR-100’s effect on tumor GBM growth, and suggest clinical potential for microRNA-related GBM therapy.

## Introduction

Glioblastoma multiforme (GBM) is the most aggressive primary human brain tumor. In the US, approximately twelve thousand new GBM patients are diagnosed annually [1], accounting for more than fifty percent of all detected malignant brain cancers and twenty percent of all primary intracranial tumors [2,3]. Even with the best standard therapies, median patient survival ranges from fourteen months to two years [4,5]. The study of cancer-associated, naturally-occurring regulatory microRNAs may lead to more effective GBM treatments. MicroRNAs are small noncoding RNAs (16-22 nucleotides) known to mediate post-transcriptional repression of protein-encoding mRNAs [6,7]. Chan et al. has demonstrated microRNA involvement in apoptosis [8]. Furthermore, microRNAs were known to regulate proliferation [9,10]. Recent reports suggest that microRNAs play a role in GBM tumorigenesis [11]. For example, miR-218 was reported to exert anti-GBM activity via NF-kB regulation [12]. Many microRNAs have been identified to affect glioma cell growth in vitro and in vivo and some are already in clinical trials [13–15]. However, the biological function of many others microRNAs are still under investigation. Patterns of differential expression of microRNAs have been demonstrated in GBM in recent reports [16,17]. In this work, microRNA profiling analysis of human GBM against human non-tumor cell lines, miR-100 was one of the top down-regulated microRNAs. Significant miR-100 down-regulation was detected in multiple patient-derived and established GBM cell lines compared to control, non-tumor brain cells, thus suggesting anti-oncogenic role for miR-100. Recently, miR-100 was reported to have anti-angiogenic function through mTOR signaling repression in endothelial cells [18]. In addition, Liu et al. displayed miR-100 as tumor suppressor and clinical marker for high tumor stage in non-small cell lung cancer [19]. In this work, the therapeutic utility of miR-100 over-expression was tested and shows reduced GBM proliferation and improved survival in a mouse xenograft model. In silico analysis showed that SMRT/NCOR2 is one of the top targets of miR-100, which was confirmed experimentally. Further analysis showed that GBM cell viability requires SMRT/NCOR2 expression. Therefore these data suggest that miR-100 has anti-tumor effect by modulating SMRT/NCOR2. 

## Materials and Methods

### Isolation of Human GBM lines

All de-identified, residual human tumor specimens were collected after surgery with patient informed consent and with approval of University of Wisconsin-Madison Institutional Review Board (Human Assurance # 00005399). Two primary serum-cultured GBM lines (22T and 33T) were derived from patients through previously reported procedures [20,21]. Both primary human lines were used in this study with two additional standard (U251 and U87) serum-cultured GBM lines. U251 and U87 GBM and control human astrocytes lines were kind gifts from Dr. Andreas Friedl (UW-Madison) [22]. Briefly, tumor tissue was collected directly from surgery, weighed, coarsely minced with a scalpel blade, and subsequently chopped twice at 200μm using a Sorvall TC-2 Smith-Farquhar tissue chopper. Chopped tissue was directly plated for growth and maintenance in suspension at 10 mg/ml in Dulbecco modified Eagle medium–low glucose, 10% FBS (Fetal Bovine Serum), and penicillin-streptomycin-amphotericin (PSA). The cells were grown and regularly split in humid 5% CO_2_ incubator cultures.

### Small RNA Isolation and Quantitative RT-PCR

All materials related to RNA isolation and amplification, such as probes and primers, were ordered from Life Technologies (formerly Invitrogen). Absolute quantitation with real time PCR (Applied Biosystem) was performed according to the recommended protocol in 20ul reactions via TaqMan assay two steps quantitative real time PCR (qRT-PCR) with reagents and probes bought from (Invitrogen). 30ng of RNAs were used per reaction, with the housekeeping 18s RNA serving as control with the ΔΔCT method. Details were described in a prior publication [23]. 

### Transfection of microRNA and siRNA

Previously published procedures were used [24,25]. microRNA-100 precursor, siRNA (silencer)/siSMRT, and control miR (non-specific microRNAs) were purchased from Life Technologies (formerly Invitrogen). All transfections were done according to the provided protocol using PepMute reagent from SignaGen Laboratories (Rockville, MD). Scramble (control miR) and miR-100 microRNAs were used at 15 pmoles per 500k cells. SMRT/NCOR2 silencers were used at 9 pmoles per 500k cells. The achieved transfection efficiency was 99% (Figure S3C).

### Cell Survival Assay

MTS assay (Cell titer 96 AQ non-radioactive cell proliferation assay from Promega, WI) was performed in 96 well plates. Three thousand cells were inoculated and starved for 24 hours. Then, miR-100 microRNA precursors were transfected. Control transfections were performed with non-specific microRNA. QPCR confirmed miR-100 overexpression in miR-100 transfected cells (Figure S3A). All assays were performed with commercially recommended protocols. 

### Cell Proliferation Assay

 Click-iT EdU (comparable to BrdU) assay was performed according to the manufacturer (Invitrogen) recommendation. Ten thousand cells were plated for 24 hours and then treated with miR-100, control miRs, or miR-100 in combination with SMRT/NCOR2 expression vector (pSMRT; Fisher Scientific).

### TUNEL Assay

Ten thousand cells were inoculated on fluorescent slides (Fisher Scientific) overnight. Transfections with miR-100, siSMRT/NCOR2 or control miR were done the next morning. TUNEL assay (Promega, WI) was performed 24 hours after transfection. Photomicrographs were taken using a fluorescence microscope (EVOS fl) with a built-in AMG computer (software version 15913).

### Luciferase Reporter Assay

The SMRT/NCOR2 3’UTR is 1052nt long and the miR100 seed sequence is located from 665 to 676nt. NIH 3T3 fibroblast and U251 cells were co-transfected with microRNA (15 pmoles) and 3’UTR luciferase reporter plasmid (1 ug) in 96 well culture plates. 24-48 hrs later, light switch luciferase assay reagent (Switch Gear Genomics, Menlo Park, CA) was added, then signal detected via Microplate Luminometer (Turner Biosystem, Inc., CA) running Veritas software version 1.9.2. Two reporter plasmids were used. One had the 3’UTR of SMRT mRNA with miR100 seed sequence, while this was mutated in the negative control.

### Tumor Xenograft Assay

Tumor xenografts were generated via implantation in NOD SCID mice via stereotactic injection according to an animal protocol approved by the UW-Madison Institutional Animal Care and Use Committee (Animal Assurance #A3368-01). Briefly, GBM cells were enzymatically dissociated to single cells, and varying cell numbers (10^6^) were suspended in 5 μl of PBS. Using a Hamilton syringe, the cells were stereotactically transplanted into the right striatum of anesthetized NOD-SCID immunodeficient mice at 0.33 μl/min at the following coordinates referenced from bregma: 0 mm anteroposterior, +2.5 mm mediolateral, and −3.5 mm dorsoventral [26]. Xenograft growth was detected and verified with animal MRI, and brains containing xenografts were obtained from animals after observed death or after euthanasia due to moribund status according to approved protocol. The humane endpoint criteria were described in our protocol as follow: During the post-operative period, animals were checked daily to ensure they can eat, drink, eliminate, and ambulate normally.  Any evidence of wound infection (drainage, swelling, redness or warmth) was brought to the attention of the veterinary staff.  If euthanasia is deemed necessary based on i.e., severe wound infection or failure to resume normal activities (ambulation, elimination), or if the animal appeared to be in pain beyond that expected as a normal surgical side-effect, (based on hunched stature, rapid respiration and not eating) cervical dislocation was performed after a state of sedation that obtained with Isoflurane, to effect, while closely monitoring respiratory rate. Once all animals have completely recovered from surgery they were evaluated for pain or discomfort and additional buprenorphine (0.05-0.1 mg/kg for mice), were administered as needed. They then were transferred to the SMPH LAR Facility in covered cages and maintained by LAR staff in aseptic conditions. Twice daily on weekdays and once daily on weekends research staff checked animals for signs of pain or discomfort and also evaluated animals for signs of neurological impairment to indicate the extent of tumor growth. Body scoring index to evaluate loss of body weight (measured every 2 days) was used, with a score of less than 2.0 being the criterion for imaging and subsequent euthanasia. Analgesia with buprenorphine was provided as needed. Neurological signs such as seizures, weakness, unsteady gait, excessive circling behavior or marked aggression or timidity, along with signs of pain or discomfort, was considered as signs of tumor progression.  Once sufficient tumor signs are noted in one animal of a group all animals in that group underwent MR imaging to evaluate the actual extent of tumor progression and decisions were made on an individual basis regarding euthanasia with veterinary staff recommendation.

### Live Animal MR Imaging

Tumor xenografts in mice were visualized by magnetic resonance imaging (MRI) using T1-weighted gadolinium enhancement in an UW approved animal protocol as previously described [27,28]. Omniscan reagent was used at the recommended ratio (W/V). Agilent (formerly Varian) 4.7T horizontal bore imaging/ spectroscopy system with VNMRJ software V3.2 was used. 

### Immunohistochemistry

Immunohistochemistry protocol was previously described [29–31]. According to approved animal protocol, mouse brains were collected at death or after euthanasia when moribund for paraffin embedding, and 5um thick tissue sections made by the UW Experimental Pathology Shared Service Translational Research Initiatives in Pathology (TRIP) lab. SMRT/NCOR2 and Ki-67 immunostaining were performed by UW Carbone Cancer Center Experimental Pathology Shared Service on sections from paraffin embedded samples, with hematoxylin counter-staining. Anti-Ki-67 (OriGene, MD) and anti-SMRT/NCOR2 (Santa Cruz Biotechnology, TX) antibodies were used at 1:200 dilution. Three random representative fields within tumor areas were used for quantitation. Each Ki-67 and SMRT/NCOR2 positive cell was counted and normalized to the total number of cells in each field.

### Immunoblot Analysis

Immunoblot analysis was performed as described [23,32]. Cell lysates were collected after sonication for 5 seconds at low intensity. Protein assays were performed (Bio-Rad) by loading 20ug of protein samples on SDS-page 10%-20% gels (Life Technologies). Transfers were performed on semi-dry transfer cell (Bio-Rad) with PVDF membranes (EMD Millipore). Anti-α -tubulin (internal control) antibody (Cell Signaling) and anti-SMRT/NCOR2 antibody (Santa Cruz) were used.

### Orthotopic microRNA injection

60 pmoles of microRNA-100 or control microRNA were injected at previously reported stereotactic coordinates used for tumor cell implantation [23,33]. MicroRNA was mixed with transfection reagent PepMute (SignaGen) in 10 μl total volume, and gradually injected (0.33 μl/min) at depths of 4mm, 3mm and 2mm. The coordinates of tumor implantation was previously recorded [23,33].

### miR-100 Expression Vector

 The following were designed and ordered from Genecopoeia (Rockville, MD): an inducible DNA construct (pEZ-lv201 backbone) containing miR-100 and GFP coding sequences, and a control vector that did not contain miR-100 coding sequence. Obtained vectors were verified with PCR and restriction analysis, and functionally tested before studies. The construct was induced with 5 ug/ml of doxycycline in culture for cell isolation purposes. The dose of induction for animals was 10 mg/kg.

### Kaplan-Meier Survival Plot

NOD-SCID mice (Jackson Laboratory, ME) were stereotactically implanted in the brain as described above with 10^6^ cells. UW Animal protocol-approved assay endpoints were the time of death or euthanasia of symptomatic animals according to recommendation of animal facility veterinarians. P-values and survival analyses were calculated based on Log-rank statistical method and presented in Kaplan-Meier plots generated using Graphpad Prism version 5.03 (Graphpad Prism Software, Inc).

### Statistics

Statistical analyses were done by Student’s *t* test, and one-way ANOVA/Tukey's multiple comparison posttests using Graphpad Prism5 (Graphpad Prism Software, Inc). . Bar graphs represent the means ± SEM (standard error of the mean) Significance level was established at (*) P < 0.05. All experiments were performed in triplicate.

## Results

### miR-100 is down-regulated in GBM

**Figure 1:**
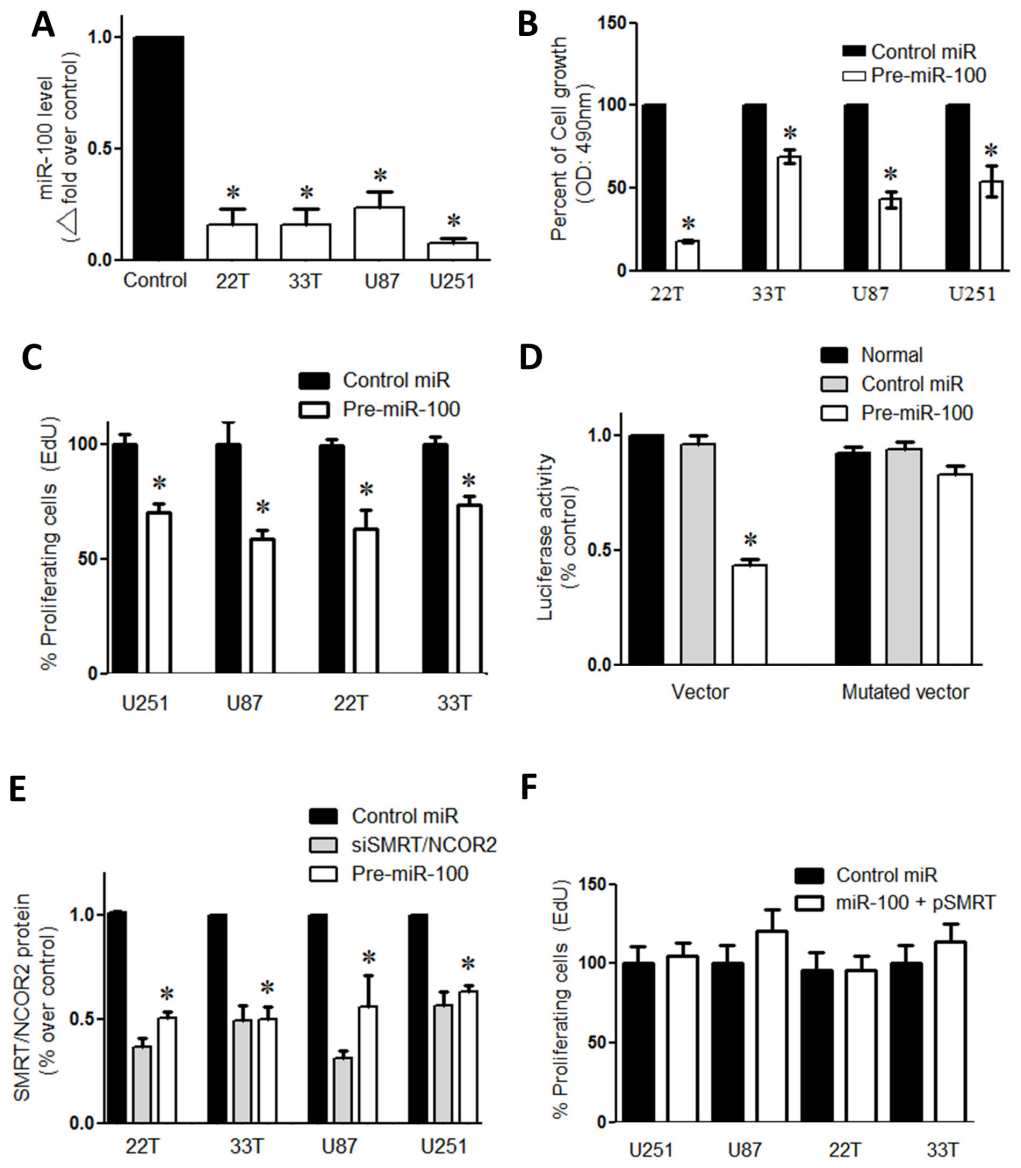
miR-100 decreases GBM proliferation and targets SMRT/NCOR2. (A) Quantitative PCR showed native miR-100 expression in multiple GBM tumor lines relative to normal control (astrocyte extracted from non-tumor brain). All GBM lines showed significantly lower miR-100 expression. (B) miR-100 overexpression decreased GBM cell growth compared to control miR transfected group. Control scrambled miRs did not affect GBM cell growth compared to miR-100. (C) miR-100 overexpression decreased GBM proliferation up to 45% compared to control miR group. (D) Transient luciferase reporter assay with either wild-type or mutated SMRT/NCOR2 3’UTR vectors. Both vectors were co-transfected with pre-miR-100 or control miRs. The normal bar represents transient luciferase reporter expression with no further treatment. (E) Western blot quantitation of SMRT/NCOR2 protein level after miR-100 expression. SMRT/NCOR2 level was reduced by transfection with either pre-miR-100 or SMRT/NCOR2 siRNAs (siSMRT/NCOR2) compared to control miR transfected group. (N=3; p ˂ 0.05). (F) EdU proliferation assay shows miR-100 overexpression along with SMRT overexpression (pSMRT) prevented proliferation inhibition reported in panel (C).

Microarray analysis showed markedly decreased miR100 expression in two independent human GBM specimens cultured in stem cell media conditions compared to human neural stem cells (hNSCs) (Supp. 1). Altered miR-100 expression was confirmed with quantitative polymerase chain reaction (qPCR) in four GBM lines (serum cultured patient-derived primary GBM lines (22T, 33T) and two standard laboratory GBM lines (U251, U87)). Compared to control astrocyte cells derived from brains free of malignancy, all four GBM lines show an average 70% lower level of miR-100 expression (P < 0.01; n=3/line; Figure 1A).

### Over-Expression of miR-100 Reduces Proliferation

Since GBM cells are highly invasive in humans and grow rapidly in culture, miR-100’s effect on GBM proliferation was tested via partially restoring miR-100 activity through transient transfection. Precursors of miR-100 (pre-miR-100) were transiently transfected into the four GBM lines (22T, 33T, U251, U87) following overnight serum starvation. After confirming miR-100 over-expression in transfected GBM (Supp. 3A), Cell numbers of miR-100 transfected cells was compared to cells transfected with control miR (non-specific microRNAs). GBM transfected with miR-100 showed 30% - 70% lower cell growth compared to controls (P < 0.05; n=3/line; Figure 1B). Proliferation was partially inhibited (30% -50%) by miR-100 overexpression compared to control miR (P < 0.05; n=3/line; Figure 1C). This is consistent with an anti-oncogenic role for miR-100 in GBM. 

### SMRT/NCOR2 Gene is targeted by miR-100

Western blot analysis of SMRT/NCOR2 – Silencing mediator of retinoic acid and thyroid hormone receptor – (also known as NCOR2) in both miR-100 transfected cells and scramble miR controls revealed an average of 50% higher SMRT/NCOR2 expression in scramble miR transfection controls (P < 0.01; n=3; Figure 1E; Supp. 4). Luciferase reporter assays were performed to assess whether miR-100 directly inhibits SMRT/NCOR2. The SMRT/NCOR2 3’UTR was linked to a luciferase reporter vector and tested in a co-transfection assay with pre-miR-100. Luciferase reporter activity was inhibited more than fifty percent (P < 0.01; n=3) in the presence of miR-100, but unaffected when the miR-100 seed sequence in the 3’UTR reporter was mutated. This reporter assay confirmed miR-100’s ability to directly inhibit SMRT/NCOR2 expression, compared to control microRNAs (P < 0.01; Figure 1D; Supp. 2A). 

### Tumor cells requires SMRT/NCOR2 activity in vitro

**Figure 2:**
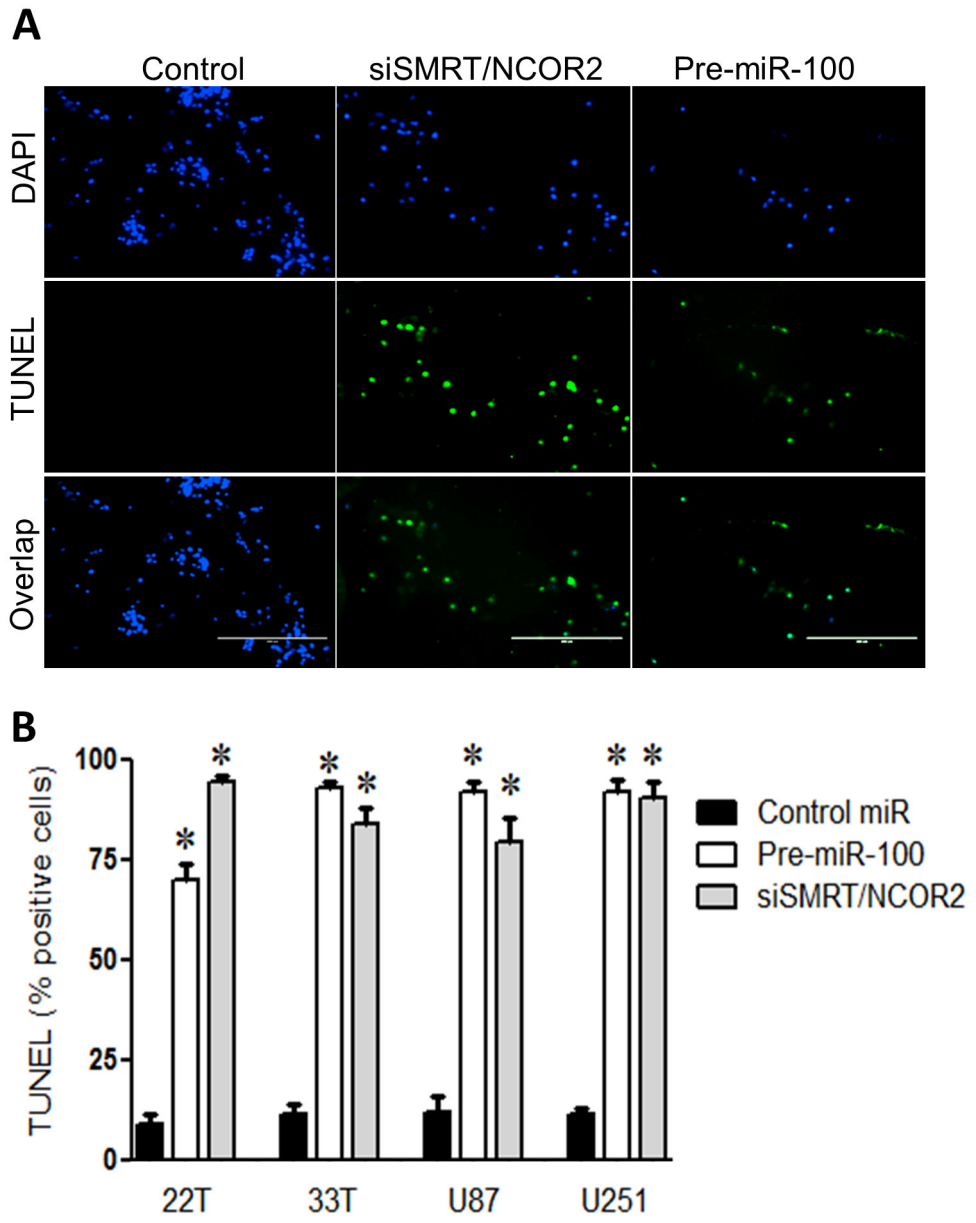
miR-100 induces cell death (A) Treatment with either pre-miR-100 or SMRT/NCOR2 siRNA (siSMRT/NCOR2) induced cell death as shown by TUNEL staining. The control cells were treated with control miR (ـــ) Scale bar, 200 um. (B) Quantitative analysis show marked increase of TUNEL-positive cells in GBMs transfected with either siSMRT/NCOR2 or pre-miR-100 compared control miR treated group.

**Figure 3:**
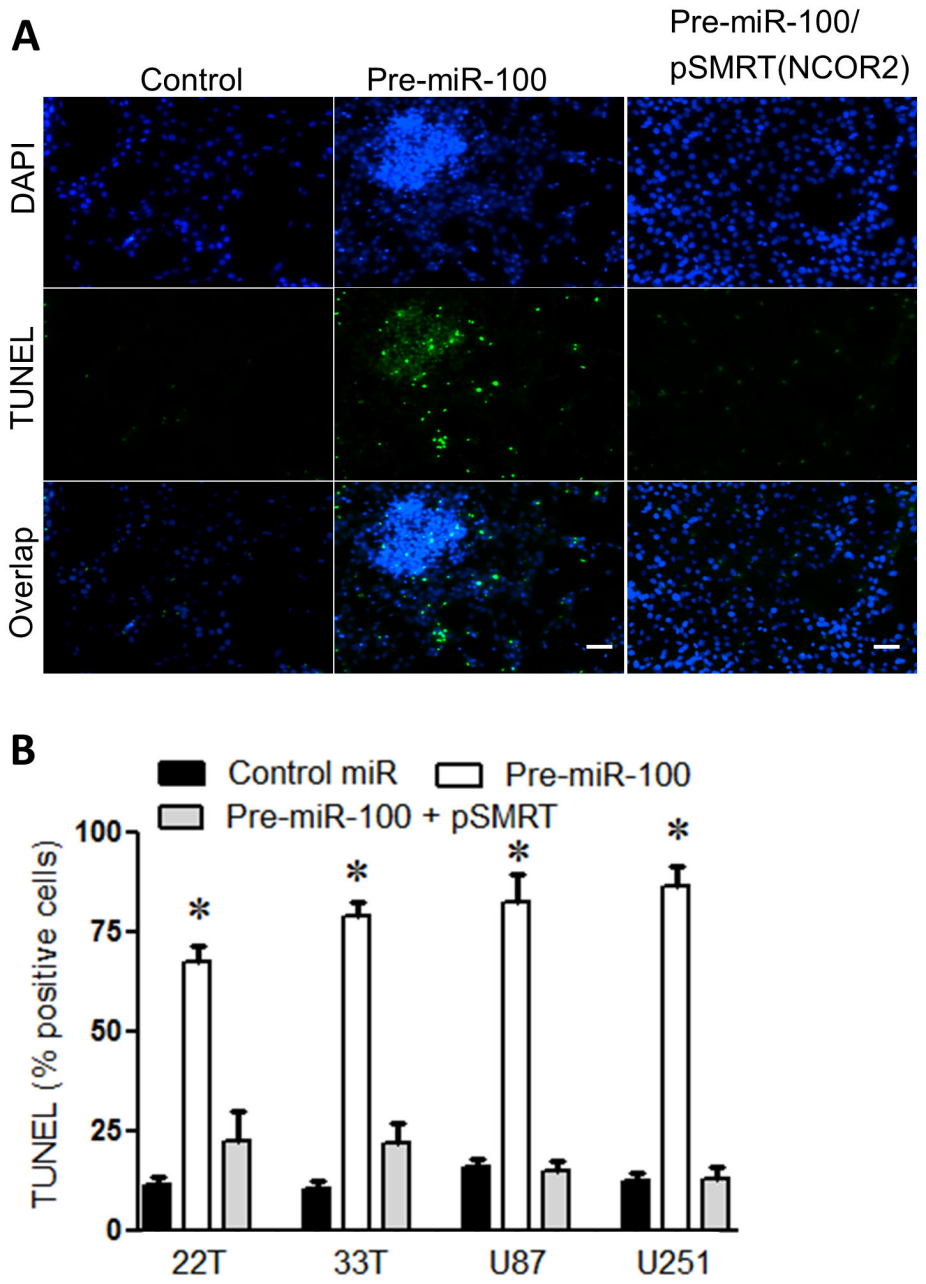
SMRT rescued tumor cells and avoids decreased cell death. (A) Treatment with both pre-miR-100 and SMRT/NCOR2 (pSMRT/NCOR2) overexpression prevented avoided induced cell death as shown by TUNEL staining compared to pre-miR-100 alone. The control cells were treated with control miR (ـــ) Scale bar, 50 um. (B) Quantitative analysis show marked decrease of TUNEL-positive cells in GBMs transfected with both SMRT/NCOR2 and pre-miR-100 similar to the level of control miR treated group.

The importance of SMRT/NCOR2 to tumor cells was interrogated by reducing its expression in vitro. siSMRT/NCOR2 (siRNAs against SMRT/NCOR2) transfection was used to abolish SMRT/NCOR2 expression. TUNEL assay measured early death in cells challenged with miR-100, siSMRT/NCOR2, and control microRNAs. More cell death was initiated in cells challenged with siSMRT/NCOR2 than controls (P < 0.01; n=3/group; Figure 2A). The same pattern was seen with miR-100 transfected cells (P < 0.01; n=3; Figure 2, A & B). Cell death has been prevented when SMRT/NCOR2 was overexpressed together with miR-100 overexpression. (P < 0.01; n=3; Figure 3, A & B).

### Ki-67 Proliferation index and SMRT are decreased in vivo

**Figure 4:**
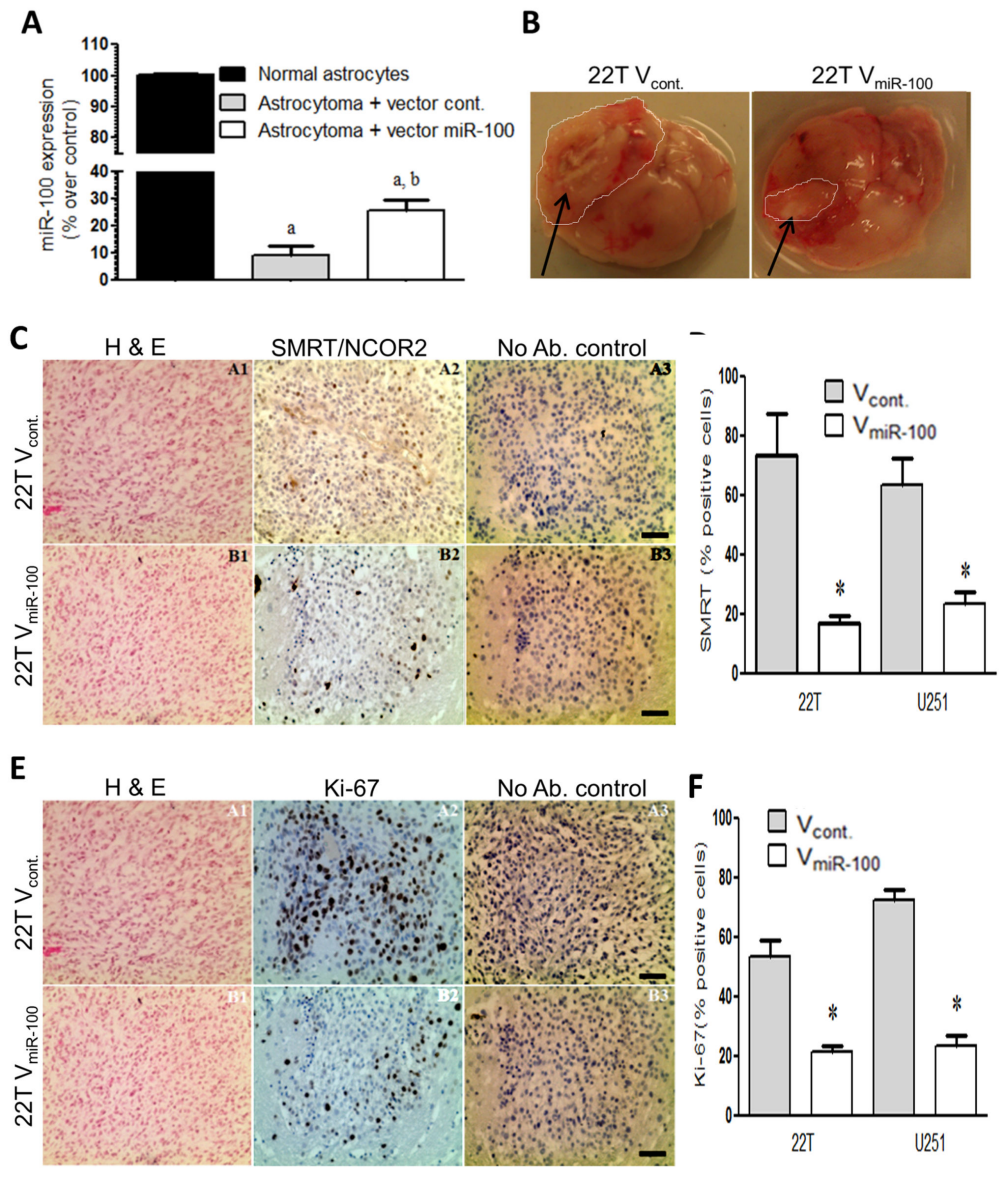
miR-100 decreases tumor size. (A) Treatment of 22T GBM cells with a miR-100 expressing vector (V_miR-100_) increased miR-100 levels by 3-fold compared to control vector (V_cont_) treated cells. Statistics: a is p < 0.05 compared to astrocyte control and b is p < 0.05 compared to V_cont_. (B) The size of the xenograft (black arrow) is larger in the V_cont_. group compared to V_miR-100_ group when brains were harvested at death or when animals were moribund post tumor implantation. Dorsal surfaces of brains and arrows to the xenografts are shown in both images. (C) A1-B1, brain section of animal from panel (B) stained with haematoxylin and eosin (H&E). A2-B2, tissue sections were immuno-labeled with SMRT/NCOR2 (brown staining). In xenografts, more SMRT/NCOR2 was seen in control (V_cont_) brain than V_miR-100_ brain. A3-B3, No primary antibody control (D) Quantitation of the sections from C shows more SMRT/NCOR2 positive cells in the V_cont_. Group over the V_miR-100_ group. (E) A1-B1, brain section of animal from panel (B) stained with H&E. A2-B2, tissue sections were immuno-stained with Ki-67 (brown staining). More Ki-67 staining was seen in control (V_cont_) brain than V_miR-100_ brain.(F) Quantitation of Ki-67 positive microscopic fields show more staining in control brains than miR-100 one. A3-B3, tissue sections were immuno-stained with no Ki-67 primary antibody. (ـــ) Scale bar, 50 um.

To test *in vivo* effects of miR-100 expression, an expression vector of tetracycline-inducible promoter linked to miR-100 and GFP genes was created. The inducible miR-100/GFP vector integrated into tumor cell genomes via lentiviral transfection. Inducible test vectors with miR-100 and GFP genes (V_miR-100_) and control vectors (V_cont._) with GFP gene only were constructed. Upon addition of doxycycline, low to medium GFP (and miR-100)-expressing cells were collected via flow cytometry (Supp. 3B), and high GFP (and miR-100)-expressing cells not used to avoid miR-100 overexpression-related apoptosis. Although GFP expression indicated vector activation, amplified expression of miR-100 was also directly confirmed quantitatively by qPCR. The isolated low-medium miR-100 expressing cell lines showed two to three-fold higher expression than in untransfected GBM cells (Figure 4A). The low-medium expressing miR-100 cells were implanted orthotopically into mouse brains to generate tumor xenografts. Then, animals were treated with doxycycline (10 mg/kg) to induce miR-100 expression from integrated vectors starting 8 days after implantation, and administered every other day at until animals were moribund or sacrificed. Mouse xenograft brain specimens (both groups harvested at equivalent times) were analyzed, grossly showing that xenografts induced for miR-100 expression were markedly reduced in growth and size (22T V_miR-100_ compared to control 22T V_cont_ samples; Figure 4B). Immunohistochemistry showed markedly lower SMRT/ NCOR2 levels in miR-100 xenografts compared to controls. 22T V_cont_ and U251 V_cont._ exhibited up to 70% and 40% more SMRT/NCOR2 label than 22T and U251 V_miR-100_ xenografts, respectively (Figure 4 C & D). In addition, Ki-67 proliferation in treated xenografts was substantially reduced compared to controls ( V_miR-100_ xenografts compared to V_cont_ controls; Figure 4E). V_miR-100_ (22T and U251) had (40% and 60%) less Ki-67 labeling, respectively, suggesting induction of miR-100 expression reduced *in vivo* tumor cell proliferation (P < 0.01; n=3/group; Figure 4F). To assess whether the doxycycline-induced three-fold increase in miR-100 expression influenced animal survival, a cohort of xenografted mice with either V_miR-100_ or V_cont_ were treated with doxycycline in a survival assay. Although a survival advantage for mice implanted with 22T V_miR-100_ was not observed (P = 248; n=8/group), we hypothesized that higher miR-100 expression (>100 fold, and closer to miR-100 expression in normal cells) may be needed to significantly improve survival.

### miR-100 reduces tumor mass and improves prognosis

**Figure 5:**
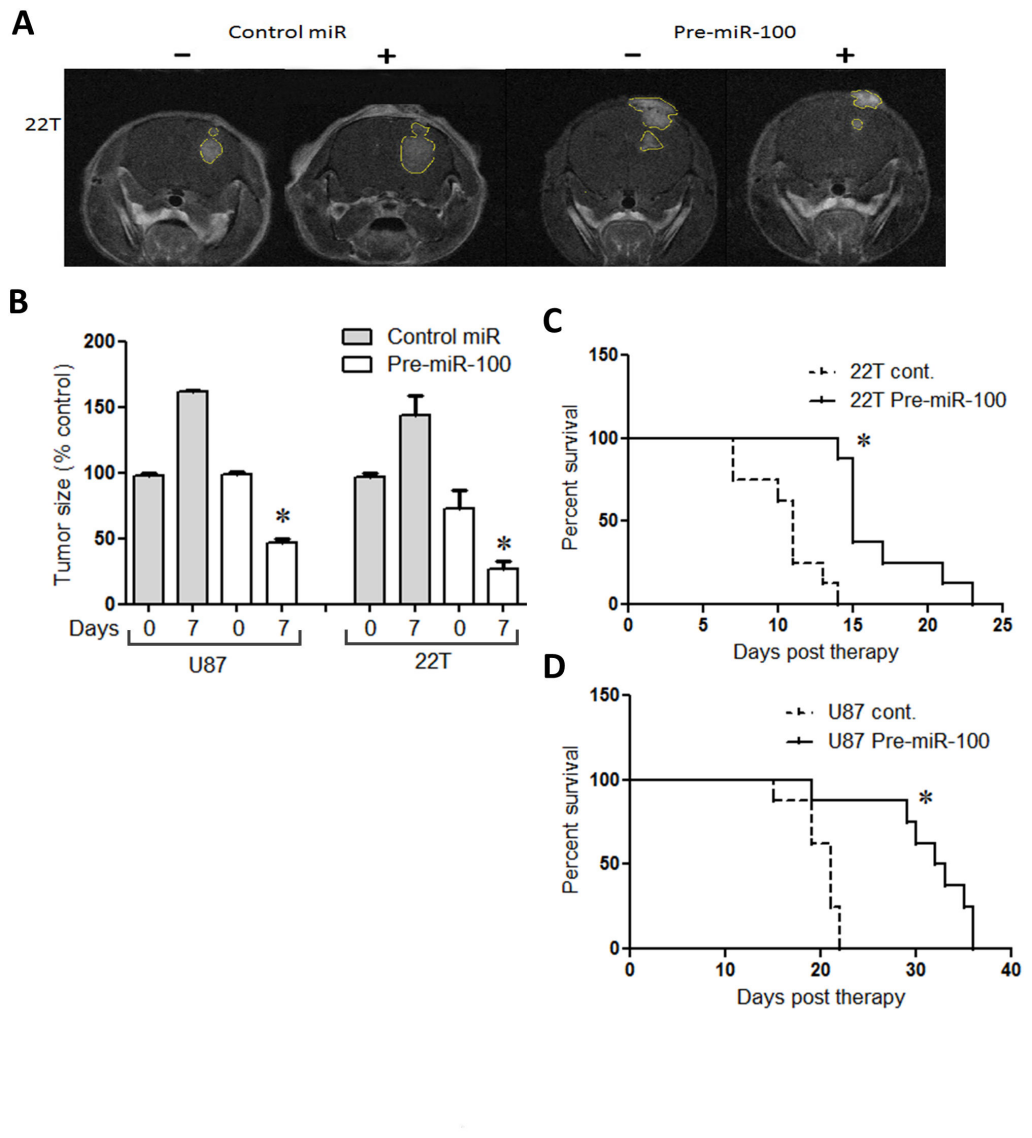
miR-100 improves survival. (A) T1 gadolinium enhanced MRI show smaller tumor xenografts after miR-100 treatment versus control miR treatment. (B) Tumor size quantitation was performed via measuring pixelation of tumor masses measured on all MRI slices per mouse and subtracted from the same mouse after treatment with miR-100 or control miR. Xenografts treated with miR-100 showed size reductions compared to continued growth of xenografts treated with control miRs (C and D) Kaplan-Meier survival analysis show that miR100-treated mice lived significantly longer than mice treated with non-specific miR (control). Survival plots correspond to Log-rank statistical method, (*) p-value < 0.05.

Experiments with single dose miR-100 injection directly into tumor xenografts were performed with two implanted GBM lines (22T and U87). After GBM implantation, mice were imaged with MRI starting at day 8. After detecting tumor xenografts at least 0.5 cm in size, 60 pmoles of miR-100 precursor or control miR were directly injected into xenografts. Tumor masses were detected and measured with MRI seven days after microRNA treatment. The miR-100 group showed significantly reduced tumor progression, while control miR treatment did not alter tumors (Figure 5A). Within one week, miR-100 treatments led to tumor size reduction by 50% compared to control miR injections (P < 0.01; n=8/gp; Figure 5B) with all mice of both groups assayed at the same timepoints. No further miR treatment was given and survival analysis showed that a single dose of pre-mir-100 extended survival 25% more than untreated controls (P < 0.01; n=8/gp; Figure 5, C & D). 

## Discussion

The therapeutic potential of miR-100 to suppress GBM proliferation and improve survival was explored in this study. Overexpression of miR-100 and specific SMRT/NCOR2 targeted inhibition relating to tumor proliferation and apoptosis *in vitro*, and *in vivo* observations of reduced tumor size and improved animal tumor model survival were shown.

microRNAs are known to be master controllers of cell metabolism [34–36]. Many microRNAs were classified as tumor suppressors or oncogenes, and targeting them through induction or inhibition was shown to result in potential therapeutic benefit [37]. The need identify or confirm new functions of miRNAs will help improve therapeutic strategies against cancer, including drug resistance. Screening microRNAs individually or even in clusters creates opportunities to identify novel predictors of drug responses.

Patient-derived GBM (22T and 33T) and standard laboratory GBM lines (U87 and U251) exhibited low endogenous miR-100 expression. In a differential microRNA profiling analysis of GBM against non-tumor cell lines, miR-100 was one of the top down-regulated miRNAs (data not shown), and QPCR assays confirmed the low endogenous miR-100 expression in all four GBM lines (Figure 1A). In lung and colon cancer studies, miR-100 dysregulation was linked to cancer progression [18,19]. Our data suggested that miR-100 acts as ‘anti-tumorigenic’ and we tested whether miR-100 over-expression reduces tumorigenicity. Transient miR-100 overexpression in all GBM lines show reduced cell growth by an average of 50% (Figure 1B). In addition, EdU assay illustrated reduced proliferation with miR-100 overexpression by 40% in all GBM lines. This is consistent with an anti-oncogenic role for miR-100 in GBM (Figure 1C). SMRT/NCOR2 (silencing mediator of retinoid or thyroid receptor) was identified *in silico* as a candidate miR-100 regulated target gene (Supp. 2A). SMRT/NCOR2 is one of the top four predicted miR-100 targets at microrna.org (Table 1). SMRT/NCOR2 activity promotes proliferation by repressing anti-proliferative genes [38]. In addition, SMRT/NCOR2 is essential for histone deacytelase 3 (HDAC3) activity that is required for chromatin structure and genome stability [11,39,40]. Furthermore, high HDAC3 expression was linked to poor prognosis in pediatric gliomas [41]. Moreover, Hercbergs et al. have proposed that silencing SMRT increases radiation sensitization of GL261 glioma [42]. In glioblastoma, Snowden et al. suggested that SMRT/NCOR2 recruitment happens when zinc finger protein mediated repression was induced [43]. Luciferase reporter assays showed that miR-100 specifically inhibited luciferase-SMRT/NCOR2 3’UTR activity, and suggested a direct relationship between SMRT/NCOR2 and miR-100 (Figure 1D). Western blot analysis showed an average 50% reduction in SMRT/NCOR2 protein level in four GBM cell lines after miR-100 overexpression, and congruent results were obtained with siSMRT/NCOR2 (Figure 1E). Simultaneous overexpression of both miR-100 and SMRT/NCOR2 eliminated proliferation bias produced by miR-100 elevation. Proliferation capacity has been restored back to the same level of control lines when SMRT vector was used (Figure 1F). Furthermore, previous reports also showed increased SMRT/NCOR2 expression in gliomas [44,45]. For example, Campos et al. have tested SMRT/NCOR2 in 283 tumor samples of astrocytic gliomas and correlated the expression with tumor differentiation, proliferation and patient survival. The study showed strong immunohistochemical staining of SMRT/NCOR2 in 93% of patient samples. The staining found within tumors’ tissue covered more than 50% of the tissue in 49.1% of the samples. This proposes an important role of SMRT/NCOR2 in glioma tumors. Therefore, SMRT/NCOR2 inhibition as a therapeutic strategy was tested. SMRT/NCOR2 was silenced via siRNA and effects on tumor cells were analyzed in culture. Interestingly, both miR-100 overexpression and SMRT/NCOR2 silencing showed increased GBM apoptosis via TUNEL assay. Both transient miR-100 over-expression and siSMRT/NCOR2 activity introduced a minimum of 70% more cell death than controls in all cell lines tested (Figure 2, A & B). Rescue of SMRT/NCOR2 inhibition by overexpressing it (with a vector) in the presence of miR-100 significantly reduced cell death up to 100% (Figure 3, A & B). Cell death detected after SMRT overexpression was statistically insignificant. This confirms the importance of SMRT/NCOR2 as a tumor modulator in GBM lines.

**Table 1:** Top 4 predicted hsa-mir-100 mRNA targets from microRNA.org.

**Candidate mRNA targets of has-miR-100**	**ID of each transcripts**	**PhastCons score**	**mirSVR score**
TMPRSS13	NM_001077263	0.65	-1.29
SMARCA5	NM_003601	0.73	-1.27
ANKAR	AK058144	0.71	-1.26
**NCOR2 (SMRT)**	**NM_006312**	**0.64**	**-1.24**

These were the top 4 out of 1389 targets predicted by microRNA.org algorithm for hsa-miR-100. The miRSVR score is the combined score for all target sites of the miRNA.

An orthotopic xenograft model was used to test in vivo miR-100 effects. Since similar patterns with all cell lines were observed in pilot experiments, we chose to focus on creating one tumor line (22T) with stable inducible miR-100 expression for animal experiments. 22T GBM-derived lines stably transfected with a doxycycline-inducible miR-100-GFP lentiviral vector were isolated that exhibit low/medium GFP expression after 24 hours of induction for further analysis (Supp. 3B). Despite the fact that isolated (low) GFP cells (V_miR-100_) had two to three-fold higher miR-100 expression than control (V_cont_) or endogenous miR-100 expression in GBM (Figure 4A), this expression level was significantly below the endogenous miR-100 level in control astrocytes (Figure 4A). Xenograft models were created by implanting modified 22T GBM cells expressing inducible low/medium miR-100-GFP, or 22T GBM cells transfected with control vector (inducible GFP only). Doxycycline was administered every other day to induce miR-100 expression starting eight days after implantation when tumor was detectable on MRI. Animals were sacrificed when neurologically compromised or moribund. Animals harboring miR-100 expressing xenografts did not have a significantly longer survival compared to the control group (p = 0.248). Inspection of mouse brains grossly after death showed larger xenografts in the control mice than tumors in the miR-100 group (Figure 4B). These *in vivo* observations were hypothesized to correlate with SMRT/NCOR2 dosage or activity. You et al reported that SMRT/NCOR2 function was required *in vivo* specifically for HDAC3 deacetylation function, although deletion of SMRT/NCOR2 did not result in embryonic lethality [11].

Jepsen et al. described SMRT/NCOR2’s role in differentiation of neural stem cells to neurons through repression of H3K27 demethylase. Although no differences in cortical progenitors derived from E13 SMRT^-/-^ (deleted) or wild type cells were observed after 6 days in culture, reduced proliferation (via Ki-67 analysis) in SMRT/NCOR2-deleted mutants were observed without changes in apoptosis [46]. Since we identified SMRT/NCOR2 as a target for miR-100 inhibition, tumor xenograft brain sections were immuno-labeled with anti-SMRT/NCOR2 and anti-Ki-67 (Figure 4, C & E). V_cont_ xenografts showed 70% more SMRT/NCOR2 staining and 40% more Ki-67 signal compared to V_miR-100_ xenografts (Figure 4, D & F). The two to three fold higher miR-100 expression (compared to endogenous GBM miR-100 expression) showed significantly reduced tumor proliferation activity, (shown by Ki-67 assay), but did not lead to a statistically significant improvement in survival (P = 0.248; n=8).

To examine the possibility that therapeutic miR-100 effects required much higher expression levels, a cohort of sixteen mice were implanted with native (22T) tumor xenografts that were then challenged with direct orthotopic injection of miR-100 or control miR. Before the challenge, mice were regularly imaged with MRI (gadolinium enhancement) until tumor xenografts were detectable (0.5 cm minimum). Seven days after stereotactic injection of microRNA-100 or control miR into xenografts, mice were imaged again to detect tumor sizes, then overall survival was measured. The miR-100-injected xenografts were smaller on MRI than the control miR-injected xenografts (Figure 5A). Matched MRI images of xenografts were quantified between both groups in order to estimate relative tumor size differences. MiR-100 injected group showed smaller tumors by 50% on average compared to control (Figure 5B). In addition, a single dose of 60 pmol of miR-100 improved survival by a mean of 20% compared to injection of scramble controls (Figure 5C). This finding was also experimentally replicated with another GBM line (U87), showing that orthotopic injection of miR-100 into brain tumors extended life around 25% compared to control (P < 0.01; n=8; Figure 5D).

Serum supplemented media is a time-tested method of culturing tumor cell lines. However, this approach has multiple limitations compared to the use of serum free growth conditions [47,48]. Specifically for glioblastoma, Lee and colleagues showed that serum-free, stem cell media isolation of human GBM cell lines yielded closer genetic fidelity to parental GBMs, and likely results in more reproducible biological properties that correlate with clinical behavior compared to serum-cultured lines from the same GBM specimens [49]. Therefore, although we first identified miR100 down-regulation via microarray analysis of GBM cells isolated via serum-free media (Figure S1), the clinical applicability of our results will be further supported if verified in planned experiments with serum-free, stem cell media cultured GBM lines.

In conclusion, this study provides strong evidence that miR-100 activity inhibits GBM tumorigenicity *in vitro* and *in vivo*. Tumor proliferation was markedly reduced and GBM apoptosis was induced by miR-100 expression above endogenous GBM levels. Stable transfected GBM cell lines harboring an inducible miR-100 expression vector (miR-100 at two-three fold higher level than endogenous GBM levels) showed marked reduction of both Ki-67 proliferation by 40%, and SMRT/NCOR2 level by 70% in tumor xenografts. Moreover, miR-100 overexpression via therapeutic tumor injection improved animal survival. Overall, we report data showing anti-tumor role for miR-100 in GBM, and reveal that regulation of miR-100 levels, plus SMRT/NCOR2 and associated downstream pathways (e.g. HDAC3) are potential therapeutic targets for GBM.

## Supporting Information

Figure S1 **Identification of altered miR-100 expression in glioblastoma.** Microarray analysis comparing miR100 expression in two independent human GBM specimens cultured in stem cell media conditions with human neural stem cells (hNSCs). TaqMan real-time PCR assays were used to confirmed miR-100 down-regulation. This heat map shows data from two independent trials.
(PDF)

Figure S2 **miR-100 predicted binding.** (A) miR-100 predicted binding to the seeding sequence of SMRT 3’UTR using microrna.org algorithm.
(TIF)

Figure S3 **Quality control of miR-100 transfection and isolation of stable miR-100 transfectants.** (A) The bars represent qPCR relative fold measurement of miR-100 level after transfecting cells with control miR or miR-100. Tumor cells were used in this assay. (B) Flow cytometry data show medium-level GFP expressing cells (GFP-mid) that were isolated for implantation, while high and low GFP expressing (GFP-high and GFP-neg) cells were discarded. (C) MicroRNA transfection efficiency was calculated at 99% as shown. (ـــ) Scale bar, 200 um.
(TIF)

Figure S4 **Western blot of reduced SMRT protein when miR-100 was over expressed.** (A) Western blot shows SMRT protein levels when cells transfected with control miRs, pre-miR-100 and siSMRT (SMRT siRNA). SMRT levels was reduced when both siSMRT and pre-miR-100 were overexpressed, n=3.
(TIF)
