# Supplementary figures and images for: microRNA-100 Targets SMRT/NCOR2, Reduces Proliferation, and Improves Survival in Glioblastoma Animal Models
Source: PLoS One. 2013 Nov 14;8(11):e80865. doi: 10.1371/journal.pone.0080865 (PMC3828259; doi:10.1371/journal.pone.0080865)

Array.data

| Trial   | hNSC | 12.1<br>CSC | 22<br>CSC |
|---------|------|-------------|-----------|
| miR-100 |      |             |           |
| miR-100 |      |             |           |

Folds

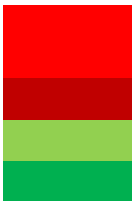

1  
0  
-1  
-2

Supplement: Figure S1 — Identification of altered miR-100 expression in glioblastoma. Microarray analysis comparing miR100 expression in two independent human GBM specimens cultured in stem cell media conditions with human neural stem cells (hNSCs). TaqMan real-time PCR assays were used to confirmed miR-100 down-regulation. This heat map shows data from two independent trials. (PDF) [file pone.0080865.s001.pdf]

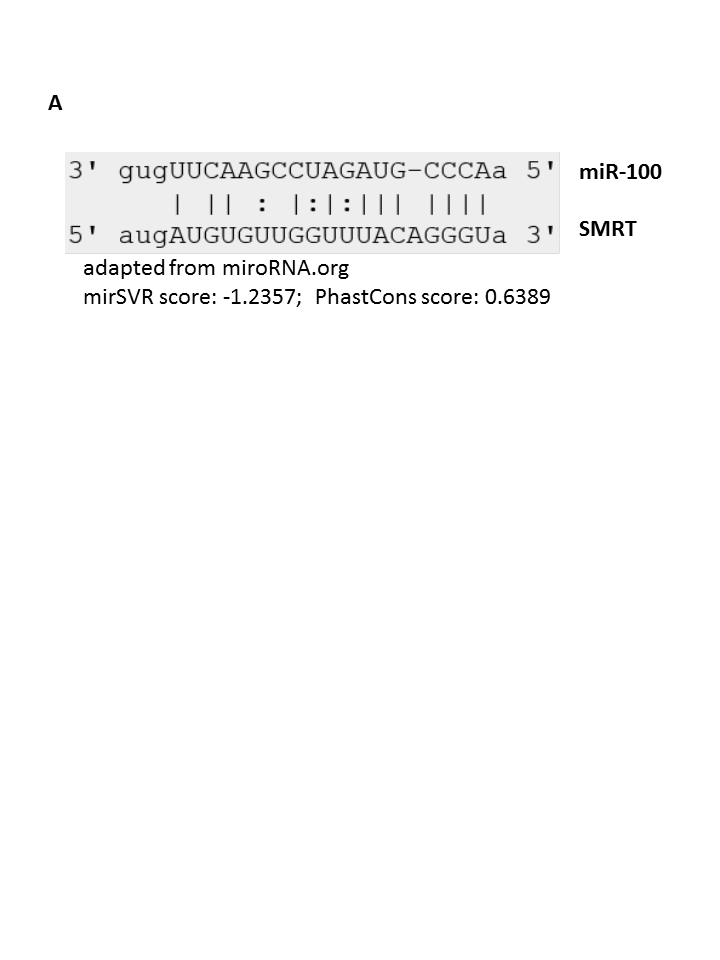

Supplement: Figure S2 — miR-100 predicted binding. (A) miR-100 predicted binding to the seeding sequence of SMRT 3’UTR using microrna.org algorithm. (TIF) [file pone.0080865.s002.tif]

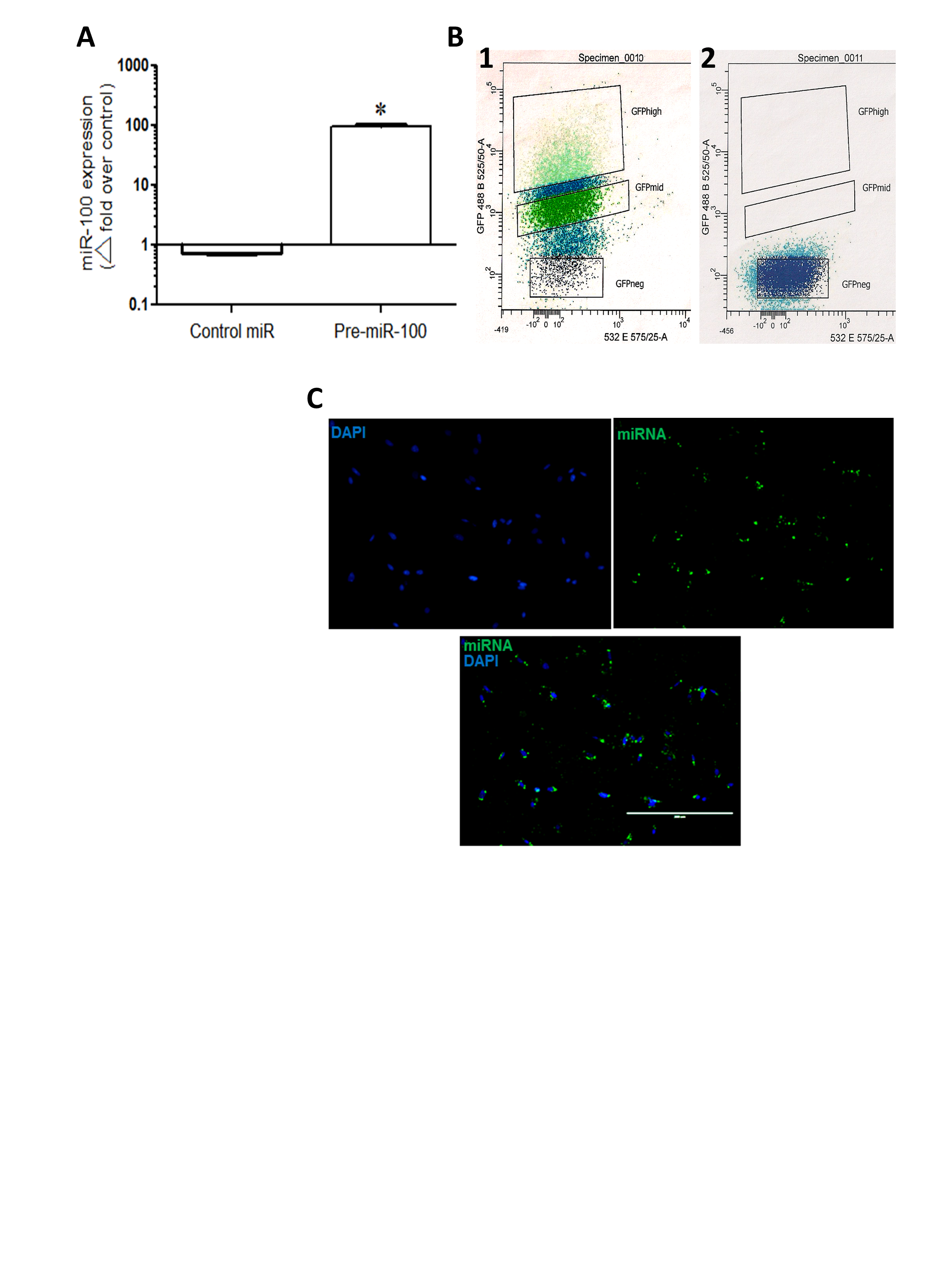

Supplement: Figure S3 — Quality control of miR-100 transfection and isolation of stable miR-100 transfectants. (A) The bars represent qPCR relative fold measurement of miR-100 level after transfecting cells with control miR or miR-100. Tumor cells were used in this assay. (B) Flow cytometry data show medium-level GFP expressing cells (GFP-mid) that were isolated for implantation, while high and low GFP expressing (GFP-high and GFP-neg) cells were discarded. (C) MicroRNA transfection efficiency was calculated at 99% as shown. (ـــ) Scale bar, 200 um. (TIF) [file pone.0080865.s003.tif]

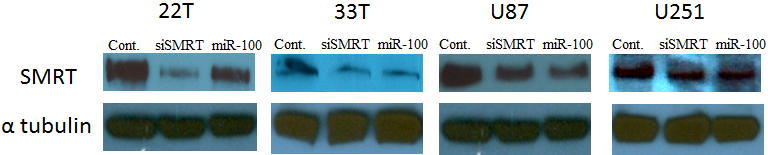

Supplement: Figure S4 — Western blot of reduced SMRT protein when miR-100 was over expressed. (A) Western blot shows SMRT protein levels when cells transfected with control miRs, pre-miR-100 and siSMRT (SMRT siRNA). SMRT levels was reduced when both siSMRT and pre-miR-100 were overexpressed, n=3. (TIF) [file pone.0080865.s004.tif]
